# Practical Guidance on Clinical Management of Belantamab Mafodotin‐Associated Ocular Events

**DOI:** 10.1002/ajh.70015

**Published:** 2025-07-28

**Authors:** Evangelos Terpos, Suzanne Trudel, María‐Victoria Mateos, Nicolás Alejandre, Kathryn Colby, Meletios A. Dimopoulos, Simona Degli Esposti, Asim V. Farooq, Maria Gavriatopoulou, Francesca Gay, Vania Hungria, K. Martin Kortüm, Xavier Leleu, Sagar Lonial, Hang Quach, Lugui Qiu, Karthik Ramasamy, Kazuhito Suzuki, Katja C. Weisel, Paul Richardson

**Affiliations:** ^1^ Department of Clinical Therapeutics School of Medicine, National and Kapodistrian University of Athens Athens Greece; ^2^ Princess Margaret Cancer Centre University Health Network Toronto Ontario Canada; ^3^ Institute of Cancer Molecular and Cellular Biology University Hospital of Salamanca/Institute of Biomedical Research of Salamanca, Salamanca University Salamanca Spain; ^4^ Department of Ophthalmology Hospital Universitario Fundación Jiménez Díaz Madrid Spain; ^5^ Department of Ophthalmology New York University Grossman School of Medicine New York New York USA; ^6^ Department of Medicine Korea University Seoul South Korea; ^7^ Moorfields Eye Hospital NHS Foundation Trust London UK; ^8^ Department of Ophthalmology and Visual Science University of Chicago Medical Center Chicago Illinois USA; ^9^ Division of Hematology University of Turin, Azienda Ospedaliero‐Universitaria Città Della Salute e Della Scienza di Torino Turin Italy; ^10^ Clínica São Germano São Paulo Brazil; ^11^ Department of Internal Medicine 2 University Hospital of Würzburg Würzburg Germany; ^12^ Service D'hématologie et Thérapie Cellulaire Hôpital La Milétrie, and Centre D'investigation Clinique INSERM 1402, Poitiers University Hospital Poitiers France; ^13^ Department of Hematology and Medical Oncology Winship Cancer Institute, Emory University Atlanta Georgia USA; ^14^ St. Vincent's Hospital Melbourne University of Melbourne Melbourne Victoria Australia; ^15^ National Clinical Research Center for Blood Diseases, State Key Laboratory of Experimental Hematology Institute of Hematology and Blood Diseases Hospital, Chinese Academy of Medical Sciences and Peking Union Medical College (PUMC) Tianjin China; ^16^ Department of Haematology, Oxford University Hospitals NHS Foundation Trust, Oxford, UK; Oxford Translational Myeloma Centre, NDORMS University of Oxford Oxford UK; ^17^ Division of Clinical Oncology/Hematology, Department of Internal Medicine The Jikei University School of Medicine Tokyo Japan; ^18^ University Medical Center Hamburg‐Eppendorf Hamburg Germany; ^19^ Jerome Lipper Multiple Myeloma Center, Division of Hematologic Malignancies, Department of Medical Oncology Dana‐Farber Cancer Institute, Harvard Medical School Boston Massachusetts USA

**Keywords:** belantamab mafodotin, clinical recommendations, multiple myeloma

## Abstract

**Importance:**

Belantamab mafodotin (belamaf)‐containing regimens are effective treatment options for patients with relapsed and/or refractory multiple myeloma. Ocular events are a common adverse effect of belamaf treatment, which can be managed by physicians, following appropriate training, to minimize their impact on the patient.

**Objective:**

To develop clinical recommendations for the identification, monitoring, and management of ocular events associated with belamaf therapy, and guidance for any required dose modification.

**Evidence Review:**

A systematic literature review was conducted and an international group of hematologists and ophthalmologists reviewed clinical trial data, real‐world evidence, and published literature on belamaf‐associated ocular events. This literature review and the collective experience and expertise of the panel of experts informed the recommendations.

**Findings:**

Belamaf‐associated ocular events are common side effects of treatment, which are managed primarily with appropriate dose modification and decreased frequency of dosing, as well as the use of artificial tears. These events affect the corneal epithelium, are mainly Grade 1 and 2 per the Keratopathy and Visual Acuity scale and are reversible in almost all patients. In general, before initiating belamaf therapy, all patients should undergo a baseline ophthalmic evaluation with an eye specialist. However, if there are delays in obtaining ophthalmic evaluation, or if a patient is rapidly progressing, treatment should be initiated, and ophthalmic evaluation should be undertaken as soon as possible. Patients should be also evaluated by an eye specialist before administering the next three belamaf doses (i.e., before Cycles 2, 3, and 4); dose modifications, as described in this paper, may apply if required. Importantly, modifying the belamaf dose in response to an ocular event is not associated with any reductions in treatment efficacy. After Cycle 4, the treating physician may use the Vision‐Related Anamnestic tool, alongside clinical judgment, to decide whether to administer the next dose of belamaf or to refer the patient to an eye specialist (i.e., if the patient experiences new worsening of vision or if the ocular events have neither improved after 8 weeks nor resolved after 12 weeks).

**Conclusions and Relevance:**

The expert panel developed recommendations for managing belamaf‐associated ocular events, with the aim of contributing to the ease of clinical use of belamaf and improving patient outcomes.

## 1 Background

Despite advances in therapeutic options and improved patient outcomes, multiple myeloma (MM) remains a challenging disease, with relapse occurring frequently [1]. Symptoms including bone pain, fatigue, and infections further contribute to disease burden and impact quality of life (QoL) [2, 3]. Patients often become refractory to multiple treatment options, leading to poor prognostic outcomes (median overall survival: 10.5–11.1 months) [1]. Belantamab mafodotin (belamaf) is a first‐in‐class, humanized antibody–drug conjugate (ADC) that binds B‐cell maturation antigen, a target expressed on malignant plasma cells in patients with MM, destroying MM cells through direct killing and immune response [4].

Ocular events are well documented in patients receiving ADCs and are common in patients receiving belamaf [5]. These ocular events are reversible in almost all patients and range from dry eye and photophobia to more serious issues impacting vision [5, 6, 7]. The exact mechanisms underlying ocular toxicity remain unknown, but increasing evidence suggests this may be off‐target toxicity driven by mechanisms such as macro/micropinocytosis (Figure S1) [8, 9]. This leads to corneal microcyst‐like epithelial changes, observed on slit lamp microscopy, which typically resolve with treatment hold [5, 9, 10, 11].

Due to the novel nature of belamaf, clear and clinically applicable guidance, training, and communication for physicians on managing these ocular events is required, including how best to modify belamaf dose (including decreased frequency of dosing) and collaboration with multidisciplinary teams (MDTs). To address this, a group of international hematologists and ophthalmologists developed recommendations for managing ocular events in patients receiving belamaf. The recommendations are informed by data from the DREAMM‐7/−8 pivotal combination therapy studies, in which a total of 398 patients with MM and at least one prior line of therapy were treated with belamaf (DREAMM‐7: *n* = 243 belamaf in combination with bortezomib and dexamethasone versus *n* = 251 daratumumab, bortezomib, and dexamethasone [median follow‐up 28.2 months]; DREAMM‐8: *n* = 155 belamaf in combination with pomalidomide and dexamethasone versus *n* = 147 pomalidomide, bortezomib, and dexamethasone [median follow‐up 22.4 versus 20.5 months, respectively]) [6, 7].

## 2 Methods

An international group comprising 16 hematologists and four ophthalmologists formed the Steering Committee (SC). The SC identified 11 clinical questions across four key themes and developed recommendations in response to each (Table 1). A systematic literature review (SLR) was conducted to evaluate relevant supporting evidence (Table S1 and Figure S2). The SC refined the recommendations over numerous rounds of review, based on the results of the SLR and their clinical experience, to ensure maximum clinical applicability of the recommendations.

**TABLE 1:** Clinical recommendations.

Theme 1: Identifying ocular conditions at baseline and ocular events in response to belamaf treatment
**Q1. What are best practices for screening, identifying, and monitoring:** **Ocular conditions at baseline?** **Ocular conditions at baseline that worsen after belamaf treatment initiation?** **New ocular events after treatment initiation with belamaf?** *Recommendation* Before initiating belamaf therapy, all patients should undergo a baseline ophthalmic evaluation with an eye specialist. However, if there are delays in obtaining ophthalmic evaluation, or if a patient is rapidly progressing, treatment should be initiated and ophthalmic evaluation should be performed as soon as possible. *For ocular conditions at baseline*: Obtain a comprehensive ophthalmologic history and evaluate for different ocular conditions (e.g., cataracts, glaucoma, etc.) Patients should be informed of potential exacerbations of pre‐existing reduced visual acuity, and ocular events, that can occur following initiation of belamaf therapy. Treatment of pre‐existing conditions is recommended according to local guidelines/practices Baseline ophthalmic evaluation should not delay treatment initiation and, in principle, baseline ocular conditions do not contraindicate belamaf treatment *After initiating belamaf therapy*: Patients should be evaluated by an eye specialist before administering the next three belamaf doses (i.e., before Cycles 2, 3 and 4). See recommendation 2 for next steps after Cycle 4 If treatment is held due to an ocular event during the first 3 cycles and the patient is symptomatic, ophthalmic examination should be performed if eye symptoms do not improve within 8 weeks or recover to Grade 1 by 12 weeks, or according to eye specialist instructions Patients should be encouraged to report further reductions in visual acuity and/or ocular symptoms to their physician (e.g., vision changes, eye pain, dry eye, photophobia, foreign body sensation) Belamaf dose adjustment may be necessary—please refer to recommendation 6
**Q2. How are belamaf‐associated ocular events followed?** *Recommendation* After the first 4 cycles of belamaf treatment, the Vision‐Related Anamnestic (VRA) questionnaire (Table 2) can be used by the treating physician to decide whether to administer the next dose of belamaf or not. *Belamaf treatment should be delayed until resolution of the ocular event if*: The patient answers ‘Yes’ to any question AND The ocular event(s) and/or impact(s) on activities of daily living are worsening AND/OR The patient has specified this has lasted for more than 8 h (especially for questions 4–9 of the VRA questionnaire) or that the symptom is persistent and does not settle spontaneously If the patient experiences new worsening of vision for more than 8 h, they should be referred to an eye specialist to ensure there are no other contributing ophthalmic conditions. For other ocular events, if these have not improved after 8 weeks or are not resolved after 12 weeks, the patient should be referred to an eye specialist, who can use the Keratopathy and Visual Acuity (KVA) scale (Table 3) to inform whether belamaf dose modification is necessary (please refer to recommendation 6 for guidance on dose modification based on KVA grading).
**Q3. Which ocular events:** **Are important to the patient?** **Are important clinically?** *Recommendation* Patients and treating physicians may prioritize different ocular events due to differing perspectives. If patients experience ocular events that affect their vision, these can interfere with daily activities (please refer to recommendation 9). However, data indicate that patients' health‐related quality of life may be less impacted by ocular events than by multiple myeloma‐associated symptoms such as fatigue and loss of appetite. Patient health‐related quality of life is also affected by bone pain, although data on the impact of this versus that of ocular events are limited. Clinically, the most important ocular events are any that require an increase in dose interval or skipping a dose cycle, although data suggest that efficacy is not compromised in this situation. In patients who are B‐cell maturation antigen‐naïve, there is evidence that early deep responses to belamaf correlate with early manifestation of ocular events. Open conversations between patients and physicians are important to ensure that both perspectives are addressed and patients feel supported.
**Q4. Are there specific patient‐ or treatment‐related factors contributing to ocular events? Can patient profiling help predict ocular events in these patients?** *Recommendation* Patients with certain baseline ocular conditions^a^ may be more susceptible to ocular events. Patient profiling can identify baseline ocular conditions that may be exacerbated by belamaf therapy but may be less useful in predicting ocular events in response to belamaf. ^a^For example, dry eye, cataracts, or glaucoma.
Theme 2: Belamaf therapy management and dose adjustment for ocular events
**Q5. What are the key indicators that would require an adjustment in belamaf dose or treatment schedule?** *Recommendation* After initiating belamaf therapy, subsequent treatment cycles should only be performed if belamaf‐induced ocular events are minimal (please refer to Table 2: The Vision‐Related Anamnestic tool). If ocular events have occurred, ophthalmic examination should be obtained to ensure these have resolved to Grade 1 before resuming treatment. Please refer to recommendation 6 for belamaf dosing guidelines. If ocular events such as those discussed in recommendation 2 develop, it is important to fully inform the patient and discuss the risks and benefits of belamaf therapy dose adjustment before proceeding (please refer to recommendation 10). Data from the DREAMM studies indicate that most patients continue to respond to belamaf therapy after dose adjustment.
**Q6. What is the most clinically effective way to adjust the dose or treatment schedule of belamaf, to both minimize relapse of multiple myeloma and manage ocular events?** *Recommendation* Please refer to recommendation 2 for classification of ocular events. Adjustment of belamaf dose or schedule in response to ocular events should be individualized (tailored to the patient's specific situation), to maximize response and mitigate the need for interruption of therapy. Please see Table 4 for recommended dose modifications following ocular events, based on ophthalmology evaluation during the first 4 cycles of belamaf.
Theme 3: Multidisciplinary collaboration for effective management of ocular events
**Q7. What are the key clinical indicators that referral to an eye specialist is necessary? What are the best practices for collaboration between hematologists and eye specialists?** *Recommendation* Per the guidance provided in recommendations 1 and 2, patients should undergo ophthalmic evaluation before administering Cycles 1–4 of belamaf therapy. After the first 4 cycles of belamaf therapy: The treating physician can use the VRA tool to decide whether to administer the next dose of belamaf or not If the patient experiences new worsening of vision that persists for more than 8 h, they should be referred to an eye specialist to ensure there are no other contributing ophthalmic conditions If in doubt or in the case of any concerns, the treating physician should use their clinical judgment to decide whether referral is appropriate (e.g., if the ocular event continues for more than 8 weeks without improvement) It is important to have effective communication between hematologists and eye specialists in all countries where belamaf is approved for use in multiple myeloma, as eye specialists may not be familiar with belamaf therapy, multiple myeloma, or both, while hematologists may be unfamiliar with managing ocular events. Constructive and streamlined working relationships between these specialists will translate into comprehensive patient‐centered care and early detection and management of ocular events, while improving patient outcomes and reducing costs.
**Q8. What educational resources and strategies are needed to improve healthcare professionals' (including eye specialists) understanding of the multiple myeloma disease course and belamaf ocular event profile?** *Recommendation* Educational strategies must be tailored to accommodate varying levels of familiarity with multiple myeloma, belamaf, and ocular events between healthcare professionals within different disciplines. Educational strategies should primarily focus on facilitating peer‐to‐peer knowledge sharing between hematologists and eye specialists, as well as between large academic centers and smaller community centers. This can be greatly facilitated through individuals having named contacts they can rely upon for guidance on complex cases. As mentioned in recommendation 7, clear communication pathways and collaboration initiatives between relevant professional specialists (including specialists with the appropriate level of specialization in a particular healthcare jurisdiction) will enhance coordinated knowledge sharing, and this should also be encouraged at a team‐member level, focusing on creating networks of communication for learning and best practice sharing. In every center, the multiple myeloma multidisciplinary team should include all relevant disciplines (such as eye specialists) either on a regular or as‐needed basis. If patients require referral to an eye specialist, appropriate follow‐up should be arranged.
Theme 4: Patient‐centric approaches in managing ocular events
**Q9. What are the primary patient concerns related to ocular events from belamaf?** *Recommendation* Belamaf‐associated ocular events may impact health‐related quality of life. Specifically, visual impairments can affect activities of daily living such as driving, reading, and using technology (television, computers, cell phones, etc.). Patients should be encouraged to report ocular symptoms during treatment to support management. Furthermore, patients should be informed that almost all belamaf‐induced visual acuity changes, which can affect activities of daily living, are reversible, according to clinical trial data (DREAMM‐7, 98%; DREAMM‐8, 92%). To address the psychosocial and emotional needs of patients, particularly those experiencing significant anxiety related to their diagnosis and treatment, patients should be reassured that support is available. Patients should be directed to any relevant resources and support groups, and this process should remain open and continuous.
**Q10. How can clinicians effectively communicate the benefits and risks of belamaf to patients to support informed decision‐making?** *Recommendation* It is important to discuss the benefits and risks of belamaf treatment transparently with patients; patients should be informed of both the expected effectiveness of belamaf therapy and the most common ocular events, how they may be affected, and management options. Using simple, nontechnical language, and visual aids should help facilitate understanding. Furthermore, the conversation should be individualized and tailored to the patient's specific situation (ie, contextualizing efficacy and risk statistics based on the patient profile as it relates to disease severity, comorbidities, baseline ocular health, etc.). Questions should be encouraged to create an open, collaborative dialogue that supports understanding and confidence to participate in shared decision‐making on the treatment plan.
**Q11. What are the key considerations for effectively communicating ocular event information to patients receiving belamaf? How can patient understanding be supported?** *Recommendation* Ocular events that can occur with belamaf treatment should be explained in full, including details on the associated symptoms and their expected onset, severity, and duration; specific instructions should be provided for what steps to take if symptoms arise. Supportive care measures, including the use of eye drops, and monitoring procedures should be explained, and reassurance given that health‐related quality of life is at the center of the treatment strategy. Furthermore, patients should be provided with contact information for support services to address any questions or concerns related to their condition or treatment. Understanding should be confirmed, for example, by asking patients to summarize what they have learned about belamaf‐associated ocular events, to ensure patient–caregiver alignment.

Abbreviations: Belamaf, belantamab mafodotin; KVA, Keratopathy and Visual Acuity; VRA, Vision‐Related Anamnestic.

## 3 Findings

### 3.1 Theme 1: Identifying Ocular Conditions at Baseline and Ocular Events in Response to Belamaf Treatment

The recommendations for this theme are presented in Table 1.

#### 3.1.1 Question 1. What Are Best Practices for Screening, Identifying, and Monitoring:

- Ocular conditions at baseline?
- Ocular conditions at baseline that worsen after belamaf treatment initiation?
- New ocular events after treatment initiation with belamaf?

Any baseline ocular condition should be documented and monitored before or soon after initiation of belamaf treatment. However, managing baseline ocular conditions should not delay timely initiation of belamaf treatment, as prompt treatment is crucial to optimize MM outcomes. Recommendations for multidisciplinary collaboration for effective management of ocular events can be found under Theme 3.

Cataracts are a common ocular condition in patients with MM, due to their prevalence in the elderly population and association with dexamethasone‐containing treatment regimens in previous lines of therapy of patients with relapsed or refractory MM. However, it is important to note that the presence of cataracts does not contraindicate belamaf treatment. The pooled prevalence of any cataract in individuals ≥ 60 years is estimated to be 54%, while the incidence of any cataract in the first or both eyes for individuals ≥ 75 years is estimated to be 70% [14, 15]. Furthermore, in a study analyzing the relationship between dexamethasone exposure and cataracts, 52% (120/231) of patients had developed cataracts at 12 months of exposure to dexamethasone [16]. Cataracts may contribute to impaired visual acuity in patients treated with belamaf, constituting a confounding factor for ocular event assessments [17]. This is supported by data from BelaRd, a phase I/II study investigating the safety and efficacy of different doses of belamaf (2.5, 1.9, and 1.4 mg/kg) in combination with lenalidomide and dexamethasone in transplant‐ineligible patients with newly diagnosed MM classified as intermediate‐fit or frail based on the International Myeloma Working Group Frailty Score (*N* = 36) [17, 18]. More patients with Grade ≥ 2 cataracts in BelaRd experienced a concurrent Grade 2–3 best corrected visual acuity (BCVA) change from baseline than those with Grade < 2 cataracts [17].

In the safety populations of DREAMM‐7 and DREAMM‐8, ocular events of any grade were more common in the belamaf arms than the control arms (DREAMM‐7, 79% versus 29%; DREAMM‐8, 89% versus 30%) [6, 7]. However, these studies show a considerable background incidence of ocular events in this patient population. Furthermore, while previous studies indicated that patients with baseline ocular conditions may be at risk of greater reduction in visual acuity following belamaf therapy than those without [11, 19], post hoc analyses of DREAMM‐7 and DREAMM‐8 showed that ocular events did not significantly differ between patients with baseline ocular conditions and those without [20]. Ocular symptoms reported during the studies (including blurred vision, foreign body sensation, and photophobia) were similar between patients with or without baseline ocular conditions (including visual acuity > 20/50, cataracts, keratopathy, or glaucoma) [20]. As with cataracts, these baseline ocular conditions do not contraindicate belamaf treatment, and the risk of greater reduction in visual acuity in patients with baseline ocular conditions receiving belamaf may be secondary to reduced baseline visual acuity.

#### 3.1.2 Question 2. How Are Belamaf‐Associated Ocular Events Followed?

The Vision‐Related Anamnestic (VRA) tool is a patient‐reported questionnaire capturing ocular symptoms (questions 1–5) and their impact on activities of daily living (questions 6–9). Table 2 presents the VRA tool, which can be used by the treating physician to decide whether to administer the next dose of belamaf. Table 3 details the Keratopathy and Visual Acuity (KVA) scale, which can be used by an eye specialist to inform whether belamaf dose modification is necessary. Figure S3 presents our proposed framework for patient monitoring and belamaf dose adjustment from Cycle 1 to Cycle 5+.

**TABLE 2:** The Vision‐Related Anamnestic tool^a^ [12].

Q1. During the last 24 h, did you ever feel that your eyes were *sensitive to light*?
Q2. During the last 24 h, did your eyes ever feel *gritty*?
Q3. During the last 24 h, did your eyes ever feel *painful or sore*?
Q4. During the last 24 h, did you ever experience *blurred vision*?
Q5. During the last 24 h, did you ever experience *poor vision*?
Q6. During the last 24 h, did you ever experience *problems in reading* due to problems with your eyes?
Q7. During the last 24 h, did you ever experience *problems in driving* due to problems with your eyes?
Q8. During the last 24 h, did you ever find it *difficult to work with a computer or a smartphone* due to problems with your eyes?
Q9. During the last 24 h, did you ever find it *difficult to watch TV* due to problems with your eyes?

*Note*: Patients are requested to answer Questions 1–9. If the patient replies ‘No’ to all of the above questions, this is considered ‘none of the time’ and treatment is continued. If the patient's reply is ‘Yes’ to any of the above questions, then the patient is requested to specify if the symptoms lasted for: 4–< 8 h (minimal); 8–< 12 h (moderate); 12–< 16 h (substantial); ≥ 16 h (severe).

Abbreviations: Belamaf, belantamab mafodotin; VRA, Vision‐Related Anamnestic.

^a^ Please refer to Recommendation 2 for specific guidance on how the VRA tool should be used to decide whether to administer the next dose of belamaf or not.

**TABLE 3:** The KVA Scale [13].

		Grade per KVA scale			
		Grade 1	Grade 2	Grade 3	Grade 4
Corneal toxicities^a^	Corneal examination finding(s)	*Mild superficial keratopathy* Mild superficial punctate keratopathy (documented worsening from baseline), with or without symptoms	*Moderate superficial keratopathy* Any/or a combination of: *Moderate* superficial punctate keratopathy *Patchy* microcyst‐like deposits *Peripheral* subepithelial haze *New peripheral* stromal opacity	*Severe superficial keratopathy* Any/or a combination of: *Severe* superficial punctate keratopathy *Diffuse* microcyst‐like deposits *involving the central cornea* *Central* subepithelial haze *New central* stromal opacity	*Corneal epithelial defect* Such as corneal erosion(s) or corneal ulcer(s)^b^
	Change in BCVA^c^	Decline from baseline of *one line* on Snellen Visual Acuity	Decline from baseline of *2–3 lines* (and Snellen Visual Acuity not worse than 20/200)	Decline from baseline by > *3 lines* (and Snellen Visual Acuity not worse than 20/200)	Snellen Visual Acuity *worse than 20/200*

Abbreviations: BCVA, best corrected visual acuity; KVA, Keratopathy and Visual Acuity.

^a^ Dose modification should be based on the most severe finding. If eyes differ in severity, the dose modification guideline should be applied based on the more severe eye.

^b^ Corneal ulcer by definition means an epithelial defect with underlying stromal infiltration.

^c^ Changes in visual acuity due to treatment‐related corneal findings.

In part 2 of BelaRd [18], the effectiveness of the VRA tool in guiding belamaf dose modifications was evaluated [12]. Patients were divided into two groups: Group A (*n* = 15), in which dosing was determined by ophthalmic exam, and Group B (*n* = 15), in which dosing was determined by patients' responses to questions in the VRA tool and the presence of Grade ≥ 3 ocular events [12]. Similar rates of Grade ≥ 3 ocular events were observed in Groups A and B, while VRA assessments for ocular symptoms and activities of daily living were also similar between both groups, supporting the effectiveness of the VRA tool [12]. Furthermore, and importantly, updated results showed no instances where hematologist‐driven belamaf dosing (using the VRA tool) was interrupted by Grade 3 ocular events [21].

It is important to identify and monitor all ocular events that impact patient QoL. Key ocular symptoms that may develop in response to belamaf treatment include visual acuity reduction, dry eye, photophobia, and foreign body sensation [7]. In DREAMM‐7 and DREAMM‐8, worsening of BCVA to 20/50 appeared to improve within 8 weeks of onset, and resolve after 12 weeks during belamaf therapy [6, 22]. Importantly, loss of efficacy has not typically been observed when belamaf delay is required, and the majority of patients experience resolution of ocular events with belamaf delay [6, 7, 23]. In DREAMM‐8, longer dosing intervals did not impact progression‐free survival (PFS); median progression‐free survival (mPFS) was not reached in belamaf, pomalidomide, and dexamethasone (BPd)‐treated patients (*N* = 93) with ≥ 1 dose delay of ≥ 12 weeks [24].

#### 3.1.3 Question 3. Which Ocular Events:

- Are important to the patient?
- Are important clinically?

Ocular events that affect vision can interfere with patients' activities of daily living such as driving and operating machinery [10]. However, analysis of patient‐reported experiences in DREAMM‐2 suggested that ocular symptoms experienced by patients treated with belamaf are mostly temporary and do not have a long‐term impact on overall QoL [25]. Patient‐reported outcome (PRO) data from DREAMM‐7 and DREAMM‐8 also indicated that QoL may be less impacted by ocular events such as blurred vision versus health‐related QoL symptoms important to patients, including fatigue and loss of appetite [3]. Furthermore, updated results from BelaRd reported patients having stopped driving or reading mainly due to eyesight issues in 0%–0.6% of monthly ocular assessments [21].

Data from DREAMM‐2, DREAMM‐7, and DREAMM‐8 indicate that belamaf dose interruptions are effective in ameliorating ocular symptoms without impacting PFS [23, 24, 26]. In DREAMM‐2, 88% (14/16) of patients continued to experience clinical benefit during the first prolonged delay (in which ≥ 3 treatment cycles were missed), 38% (6/16) had a deepened response during the delay, and 38% (6/16) maintained the same response [26]. In a post hoc analysis of DREAMM‐7, mPFS was 36.6 months in patients treated with belamaf, bortezomib, and dexamethasone (BVd) with ≥ 1 dose delay of ≥ 12 weeks (*N* = 126), compared with 36.6 months in the intention‐to‐treat population (*n* = 243) [6, 23]. Furthermore, 95% of patients with less than or equal to partial response before the first extended dose delay (≥ 2 cycles) following one to two doses reached, maintained, or deepened their response, while 95% of patients with greater than or equal to very good partial response experiencing an extended dose delay at any time maintained or deepened their response [23]. In DREAMM‐8, longer dosing intervals did not impact PFS; mPFS was not reached in BPd‐treated patients (*N* = 93) with ≥ 1 dose delay of ≥ 12 weeks [24].

In a post hoc analysis of BelaRd, from the total analysis population who completed three treatment cycles (*N* = 65), two subgroups were created based on severity and timing of ocular events; Group 1 comprised patients with Grade 2–4 ocular events until 3 months (*n* = 19), while patients with Grade 0–1 ocular events until 3 months formed Group 2 (*n* = 46) [27]. The median (months, 95% CI) times to complete response or better were 11.76 (4.37 to 13.90) in Group 1 and 23.29 (8.67 to not reached) in Group 2 [27]. Most patients (52/65; 80%) were switched to a schedule of every 12 weeks due to ocular events and, by the clinical cut‐off date, 68% and 37% of Group 1 and Group 2 patients had achieved a complete response, respectively [28]. While caution should be used when interpreting the trend between moderately severe ocular events and deep responses, this relationship warrants further investigation in larger samples [27].

#### 3.1.4 Question 4. Are There Specific Patient‐ or Treatment‐Related Factors Contributing to Ocular Events? Can Patient Profiling Help Predict Ocular Events in These Patients?

In DREAMM‐2, 19% of patients reported a history of dry eye prior to belamaf treatment and were statistically more prone to corneal events versus. patients with no history of dry eye (*p* = 0.02) [11]; we recommend that belamaf administration should take place after consultation with an eye specialist if a patient is experiencing severe dry eye. In DREAMM‐7 and DREAMM‐8, ocular symptoms reported during the studies (including blurred vision, foreign body sensation, and photophobia) were similar between patients with or without baseline ocular conditions (including visual acuity > 20/50, cataracts, keratopathy, or glaucoma) [20].

## 4 Theme 2: Belamaf Therapy Management and Dose Adjustment for Ocular Events

The recommendations for this theme are presented in Table 1.

### 4.1 Question 5. What Are the Key Indicators That Would Require an Adjustment in Belamaf Dose or Treatment Schedule?

A retrospective analysis of 35 patients treated with belamaf and dexamethasone at a single institution identified Grade 2 keratopathy, per the KVA scale, as the threshold to delay or reduce belamaf dose [29]. As mentioned under Question 3, DREAMM‐7 and DREAMM‐8 demonstrated that mPFS was not negatively impacted by dose delays, with patients often experiencing a maintained or deepened response [24, 30].

### 4.2 Question 6. What Is the Most Clinically Effective Way to Adjust the Dose or Treatment Schedule of Belamaf, to Both Minimize Relapse of MM and Manage Ocular Events?

Belamaf dosing should proceed according to the prescribing information: in combination with bortezomib and dexamethasone (BVd), 2.5 mg/kg once every 3 weeks (as used in DREAMM‐7); in combination with pomalidomide and dexamethasone (BPd; as used in DREAMM‐8), 2.5 mg/kg (Cycle 1, Day 1) followed by 1.9 mg/kg (Day 1 of every 4‐week cycle) [6, 7, 31]. Recommended belamaf dose modifications following ocular events are presented in Table 4.

**TABLE 4:** Recommended belamaf dose modifications following ocular events as defined by the KVA scale.

Eye examination findings per KVA scale		Recommended dose modifications
Grade 1	Corneal examination finding(s) Mild superficial punctate keratopathy^a^ Change in BCVA^b^ Decline from baseline of one line on Snellen Visual Acuity	Continue according to belamaf prescribing information Ophthalmic evaluation may be planned to confirm ocular event(s) do not worsen
Grade 2	Corneal examination finding(s) Moderate superficial punctate keratopathy^c^ Change in BCVA^b^ Decline from baseline of two or three lines (and Snellen Visual Acuity not worse than 20/200)	Use Q8W dosing and maintain at new dose interval, provided recovery to Grade 1 If ocular events remain Grade 2 after 8 weeks OR Grade 2 ocular events recur after initial recovery to Grade 1, consider extending dose interval to 12 weeks If dose interval exceeds 12 weeks, reduce belamaf dose^e^
Grade 3	Corneal examination finding(s) Severe superficial punctate keratopathy^d^ Change in BCVA^b^ Decline from baseline by more than three lines (and Snellen Visual Acuity not worse than 20/200)	Reduce belamaf dose^e^ AND extend dose interval to at least 12 weeks and maintain at new dose interval, provided recovery to Grade 1 If dose interval exceeds 16 weeks, further reduce belamaf dose^e^
Grade 4	Corneal examination finding(s) Corneal epithelial defect Change in BCVA^b^ Snellen Visual Acuity worse than 20/200	Consider treatment hold until Grade 1 If continuing treatment with belamaf is being considered, reduce belamaf dose^e^ AND extend dose interval to at least 12 weeks and maintain at new dose interval

Abbreviations: BCVA, best corrected visual acuity; belamaf, belantamab mafodotin; KVA, Keratopathy and Visual Acuity; Q8W, every 8 weeks.

^a^ Mild superficial punctate keratopathy (documented worsening from baseline), with or without symptoms.

^b^ Changes in visual acuity due to treatment‐related corneal findings.

^c^ Moderate superficial punctate keratopathy with or without patchy microcyst‐like deposits, peripheral sub‐epithelial haze (peripheral), or a new peripheral stromal opacity.

^d^ Severe superficial punctate keratopathy with or without diffuse microcyst‐like deposits involving the central cornea, sub‐epithelial haze (central), or a new central stromal opacity.

^e^ There are limited data on the efficacy of belamaf at doses lower than 1.9 mg/kg; if possible, the administration of doses below 1.9 mg/kg should be avoided.

Both DREAMM‐7 and DREAMM‐8 permitted dose delays/reductions to manage ocular events [6, 7]. In DREAMM‐7, the average dose intensity was equivalent to 1.9 mg/kg every 6 weeks during Months 6 to 12, and every 6 to 9 weeks during Months 12 to 18 [20]. In DREAMM‐8, the average dose intensity was equivalent to 1.9 mg/kg every 12 weeks from Months 6–12 onwards [20]. The most commonly administered dose, by dose level, in DREAMM‐7 was 1.9 mg/kg [23]. In both studies, the most common ocular event was blurred vision [6, 7]. In DREAMM‐7, 34% (82/242) of patients experienced worsening of BCVA to 20/50 in both eyes from normal BCVA at baseline (defined as 20/25 or better in at least one eye), and the median time from onset of first event to resolution was 64 days [6]. In the 2% (5/242) of patients who experienced worsening of BCVA to 20/200, the median time from onset of first event to resolution was 87 days [6]. Activities of daily living, such as reading or watching television, are increasingly affected by decreasing visual acuity; the World Health Organization defines a BCVA of 20/50 as near normal visual impairment, while 20/200 is defined as severe visual impairment and legal blindness [32].

Data suggest Grade 4 ocular events per the KVA scale (Table 3) are rare in patients treated with belamaf; in the ALGONQUIN study, one patient experienced Grade 4 BCVA worsening following treatment with 3.4 mg/kg belamaf [33].

## 5 Theme 3: Multidisciplinary Collaboration for Effective Management of Ocular Events

The recommendations for this theme are presented in Table 1.

### 5.1 Question 7. What Are the Key Clinical Indicators That Referral to an Eye Specialist Is Necessary? What Are the Best Practices for Collaboration Between Hematologists and Eye Specialists?

Supportive care, including ophthalmic examinations, should proceed according to the belamaf prescribing information. Eye evaluation by an eye specialist is required before belamaf administration at baseline and before Cycles 2, 3, and 4. After Cycle 4, the treating physician may use the VRA tool (Table 2) to decide whether to administer the next dose of belamaf or refer the patient to an eye specialist (Figure S3). The eye specialist can provide the hematologist with an examination report including the key parameters—worst grade for any corneal examination findings and any BCVA decline from baseline. The recommended dose modification is based on the worst finding (based on corneal examination finding or change in visual acuity per KVA scale) [10, 11]. Treating physicians should work closely with eye specialists to assess Grade 4 ocular events and make therapy management decisions in these cases, balancing the indication for belamaf treatment hold against the importance of continued treatment. While belamaf‐associated ocular events resolve in most cases with treatment hold [5, 6, 7], physicians should discuss the benefits and risks of treatment hold with patients, as continued treatment may be warranted despite the risks.

Clear and standardized communication pathways between hematologists and eye specialists must be established to prevent potential gaps in patient care. By actively implementing practical tools, such as reporting templates and shared electronic records, physicians can enhance information exchange and contribute further to improved patient outcomes.

### 5.2 Question 8. What Educational Resources and Strategies Are Needed to Improve Healthcare Professionals' (HCPs; Including Eye Specialists) Understanding of the MM Disease Course and Belamaf Ocular Event Profile?

HCPs' understanding of the MM disease course and the belamaf ocular event profile will vary. Ongoing collaboration and communication between hematologists/oncologists and eye‐care professionals is essential to manage ocular events and ensure patients receive optimal treatment [10]. When hematologists/oncologists prescribe belamaf, they should educate themselves and the wider MM patient‐care team on the risks of ocular events and how to manage these [10].

Structured training for both eye specialists and hematologists is required to improve consistency in the assessment of ocular events, including clear protocols and educational tools such as online modules, decision aids, case studies, and checklists. These resources would support general clinicians in community‐ or resource‐constrained settings, where access to trained eye specialists may be limited. In such contexts, equipping general clinicians to recognize and manage belamaf‐associated ocular events will help ensure timely and effective care.

## 6 Theme 4: Patient‐Centric Approaches in Managing Ocular Events

The recommendations for this theme are presented in Table 1.

### 6.1 Question 9. What Are the Primary Patient Concerns Related to Ocular Events From Belamaf?

As mentioned under Question 3, ocular symptoms experienced by patients in DREAMM‐2 did not appear to affect overall QoL long term [25]. In DREAMM‐7, patients in the BVd arm and patients in the daratumumab, bortezomib, and dexamethasone arm were comparable in terms of overall QoL, role functioning, and physical functioning [34]. Furthermore, as mentioned under Question 3, data from both DREAMM‐7 and DREAMM‐8 suggest patients are more affected by health‐related QoL symptoms such as fatigue and loss of appetite than ocular events [3]. Patients' health‐related QoL is also highly impacted by bone pain [35], although the impact of this versus that of ocular events has not been studied. In DREAMM‐7 and DREAMM‐8, almost all belamaf‐induced visual acuity changes improved, where improvement was defined as a BCVA of better than 20/50 or 20/200 (depending on the level of worsening) in both eyes [6, 7]. In DREAMM‐7, BCVA improved after the first occurrence of worsening in 98% (80/82) of patients who experienced a decrease in BCVA to 20/50 [6]. Similarly, in DREAMM‐8, BCVA improved after the first occurrence of worsening in 92% (47/51) of such patients [7]. In the remaining patients for both studies, follow‐up concluded with the first event having not improved [6, 7].

### 6.2 Question 10. How Can Clinicians Effectively Communicate the Benefits and Risks of Belamaf to Patients to Support Informed Decision‐Making?

Effective patient communication is a key aspect of medical practice—PRO data suggest that clear doctor–patient communication is associated with patient satisfaction, accurate diagnosis, and adherence to treatment [36]. Our recommendation focuses on communicating the benefits and risks of belamaf treatment, aiming to support shared decision‐making and achieve the best patient outcomes. Patients must be informed of expected ocular events, but reassured that they resolve in most cases with treatment hold [5, 6, 7].

### 6.3 Question 11. What Are the Key Considerations for Effectively Communicating Ocular Event Information to Patients Receiving Belamaf? How Can Patient Understanding Be Supported?

Patients should be informed, by referral to an eye care professional, of potential adverse events and the importance of reporting ocular symptoms [10]. Educational materials for patients and the patient care team are available through the Risk Evaluation and Mitigation Strategy program in the USA and the Risk Management Plan in the EU [10].

## 7 Interpretation

Belamaf has demonstrated substantial efficacy and a positive benefit–risk profile in patients with relapsed or refractory MM. However, ocular events are a known risk with treatment and must be monitored and managed appropriately. This can be challenging due to the novel nature of belamaf and limited available guidance around ocular events. Effectively managing the side effects of new therapies is essential to their successful translation from clinical trials into everyday MM management, including for patients with intermediate/frail status [37, 38, 39]. The SC provided recommendations on identifying and monitoring ocular events, managing these through dose modifications, and collaborating with an MDT to ensure optimal patient care. It was critical to capture both hematologist and ophthalmologist perspectives in these recommendations to provide a well‐rounded view, similar to other comprehensive efforts in specific treatment‐related toxicity management for MM patients, such as bortezomib‐induced peripheral neuropathy [40]. These recommendations aim to provide HCPs with a framework for managing ocular events with belamaf to ensure these are front‐of‐mind from treatment onset, with the ultimate aim to optimize patient management.

## Author Contributions

All authors contributed equally to the manuscript, read, and approved the final manuscript. E.T. and P.R. served as co‐chairs of the program. S.T. and M.‐V.M. led data discussions with the rest of the Steering Committee.

## Ethics Statement

The authors have nothing to report.

## Conflicts of Interest

E.T., Advisory boards: Amgen, AstraZeneca, EUSA Pharma, BMS, GSK, J&J, Menarini/Stemline, Pfizer, Sanofi, Takeda. Honoraria: Amgen, Antengene, AstraZeneca, EUSA Pharma, BMS, Forus, GSK, J&J, Menarini/Stemline, Novartis, Pfizer, Sanofi, Swixx, Takeda. Research funding: Amgen, GSK, J&J, Sanofi, Takeda. Travel expenses: Takeda; S.T., Grants: Janssen, GSK, BMS, Pfizer, K36 Therapeutics, Roche, Genentech. Consultancy: GSK, Roche, BMS. Honoraria: GSK, Janssen, Pfizer, AstraZeneca, Kite; M.‐V.M., Honoraria: J&J, BMS, Sanofi, GSK, Pfizer, Roche, Kite, Menarini/Stemline, Oncopeptides, Regeneron; N.A., Advisory board and honorarium: GSK; K.C., Honorarium: GSK; M.A.D., Advisory boards and satellite symposia: Amgen, Sanofi, Regeneron, Menarini, Takeda, GSK, BMS, Janssen, BeiGene, Swixx, AstraZeneca. Honorarium: GSK; S.D.E., Advisory boards: GSK, Annexon Biosciences, Roche UK. Speaker's fee: Bayer UK. Travel support: Bayer UK, Roche UK. Consultancy and honorarium: GSK; A.V.F., GSK, Amgen, Ambrx, Sanofi, Seagen, Eisai, AstraZeneca (Data Monitoring Committee), Skye Bioscience, Mythic Therapeutics; M.G., Honoraria: Menarini, Janssen, Integris Pharma, Takeda, GSK, Innovis, Teva, AbbVie; F.G., Honoraria and advisory boards: Janssen, BMS, Celgene, Takeda, GSK, Roche, AbbVie, Pfizer, AstraZeneca, Kite, Amgen, Menarini; V.H., Honoraria: BMS, GSK, J&J, Regeneron, Sanofi, Takeda; K.M.K., Consultancy and honoraria: BMS, GSK, Janssen, Pfizer, Sanofi, Menarini/Stemline, Takeda. Research funding: Janssen; X.L., Consultancy and honoraria: Janssen, Sanofi, AbbVie, Roche, Amgen, Novartis, Kite, BMS, GSK, Takeda, Oncopeptide, Pfizer; S.L., Advisory boards: Takeda, Amgen, Novartis, BMS, GSK, AbbVie, Genentech, Pfizer, Regeneron, Janssen, BMS. Research support for clinical trials: Novartis, BMS, Janssen, Takeda. Board of directors with stock: TG Therapeutics (neurology and autoimmune indications). Honorarium: GSK; H.Q., Research funding: AbbVie, BMS, GSK. Advisory board: BMS, AbbVie, GSK, J&J, Pfizer, Antengene, Sanofi. Honorarium: GSK; L.Q., Consulting fees, payment or honoraria for lectures, presentations, speakers' bureaus, manuscript writing, or educational events: BeiGene, AbbVie, Takeda, Janssen, Roche, Sanofi, GSK. Support for attending meetings and/or travel: BeiGene, Janssen, Takeda, Roche. Participation on a data safety monitoring board or advisory board: BeiGene, Janssen, Takeda, Sanofi; K.R., Advisory board and honoraria: AbbVie, Amgen, Adaptive Biotechnologies, BMS/Celgene, EUSA Pharma, GSK, J&J, Karyopharm Therapeutics, Menarini/Stemline, Pfizer, Sanofi, Takeda, Recordati, CellCentric, Oncopeptides; K.S., Honorarium: GSK; K.C.W., Advisory role or expert testimony: AbbVie, Adaptive, Amgen, AstraZeneca, BeiGene, BMS, Janssen, GSK, Karyopharm Therapeutics, Oncopeptides, Pfizer, Regeneron, Roche Pharma, Sanofi, Menarini/Stemline, Takeda. Honoraria: AbbVie, Adaptive, Amgen, AstraZeneca, BeiGene, BMS, Janssen, GSK, Karyopharm Therapeutics, Novartis, Oncopeptides, Pfizer, Regeneron, Roche Pharma, Sanofi, Menarini/Stemline, Takeda. Research funding: Amgen, BMS/Celgene, Janssen, Sanofi, GSK, AbbVie; P.R., Consulting: BMS/Celgene, GSK, Karyopharm Therapeutics, Oncopeptides, Regeneron, Sanofi. Research funding: Oncopeptides. Honorarium: GSK.

## Supporting information


Data S1.

## Data Availability

The authors have nothing to report.
