# Supplementary material for: Practical Guidance on Clinical Management of Belantamab Mafodotin‐Associated Ocular Events
Source: Am J Hematol. 2025 Jul 28;100(10):1839–50. doi: 10.1002/ajh.70015 (PMC12417747; doi:10.1002/ajh.70015)
Supplement: Supplementary file 1 — Data S1. [file AJH-100-1839-s001.docx]

### **SUPPLEMENTAL ONLINE CONTENT**

**Practical Guidance on Clinical Management of Belantamab Mafodotin-Associated Ocular Events**

Correspondence to: Evangelos Terpos; eterpos@hotmail.com; eterpos@med.uoa.gr

**CONTENTS**

**eTable 1.** Eligibility criteria for the identification of relevant studies

**eFigure 1.** Potential mechanism of off-target ocular toxicity with belamaf

**eFigure 2.** Systematic literature review methodology

**eFigure 3.** Patient monitoring and belamaf dose adjustment from Cycle 1 to Cycle 5+

**eTable 1.** Eligibility criteria for the identification of relevant studies

| **Criteria** | **Inclusion criteria** | **Exclusion criteria** |
| --- | --- | --- |
| **Population** | Patients with MM | Patients without MM |
| **Intervention/comparator** | Belamaf-based monotherapy or combination therapy | Any treatment without belamaf |
| **Outcomes** | 1. Identifying ocular conditions at baseline and OEs in response to belamaf treatment    - Best practices for screening, identifying, and monitoring    - Clinically important OEs    - OEs important to patient    - Classification of belamaf-associated OEs    - Susceptible subgroups    - Ocular symptoms    - Prediction of OEs 2. Belamaf therapy management and dose adjustment for ocular events    - Baseline ocular conditions    - Indicators for an adjustment in belamaf dose or treatment schedule, or both    - Effective approaches to adjust the dose or treatment schedule 3. Multidisciplinary collaboration for effective management of OEs    - Indicators for referral to an eye specialist    - Approaches to improve healthcare professionals’ understanding of MM disease course and belamaf-associated OEs 4. Patient-centric approaches in managing OEs    - Patient concerns regarding OEs    - Role of clinicians to support informed decision-making    - Key considerations for effective communication on information about OEs | Outcomes other than listed |
| **Study design** | - Clinical trials - Observational studies (eg, cohort studies, case–control studies) - Real-world evidence - Case series (≥10 cases) | - Preclinical studies - Animal/in vitro studies - Phase I - Case reports |
| **Timeframe** | 2019–2024 | Pre-2019 |

Abbreviations: belamaf, belantamab mafodotin; MM, multiple myeloma; OE, ocular event.

**eFigure 1.** Potential mechanism of off-target ocular toxicity with belamaf


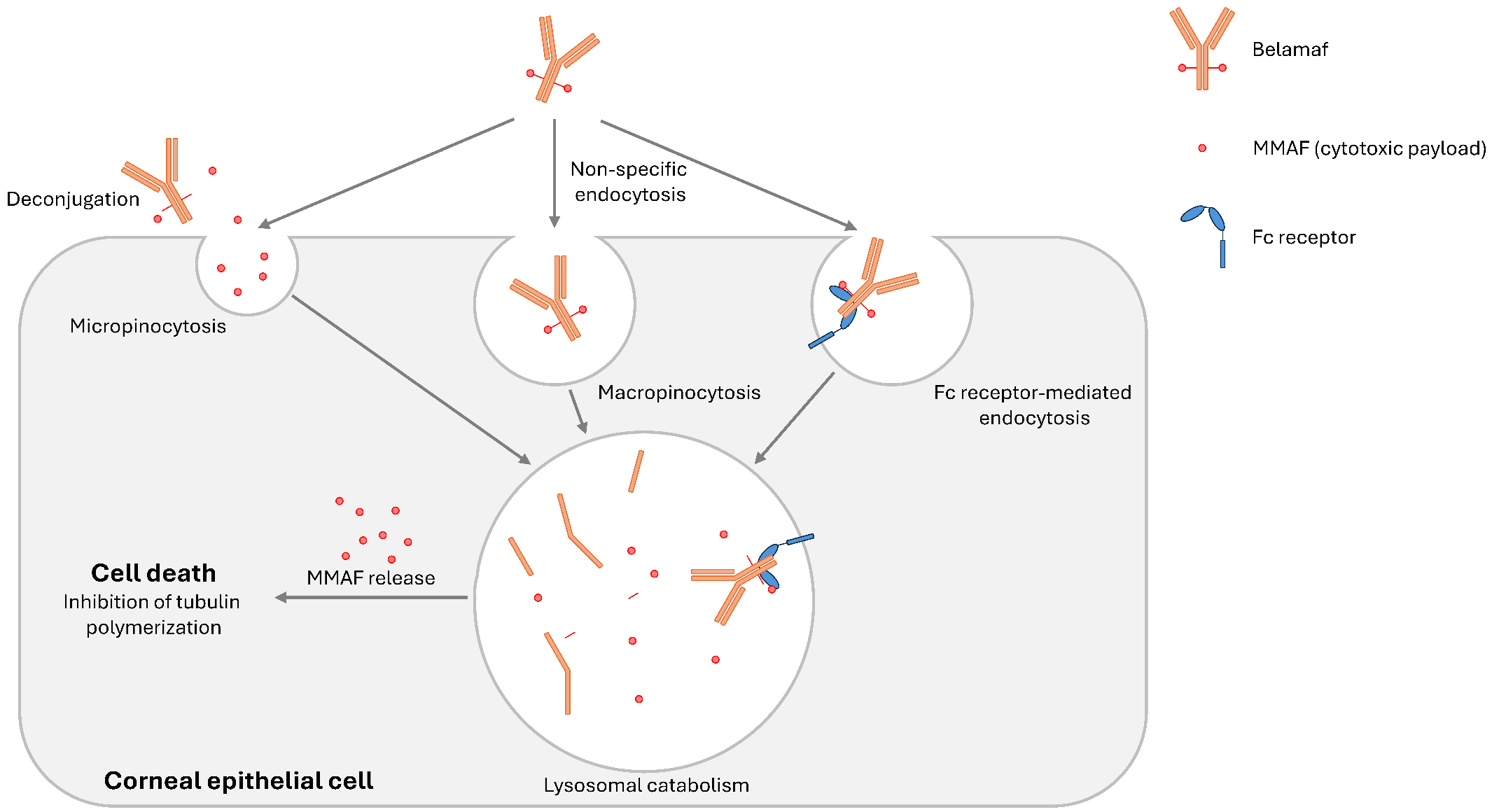


Belamaf, belantamab mafodotin; Fc, fragment crystallizable; MMAF, monomethyl auristatin F.

**eFigure 2.** Systematic literature review methodology


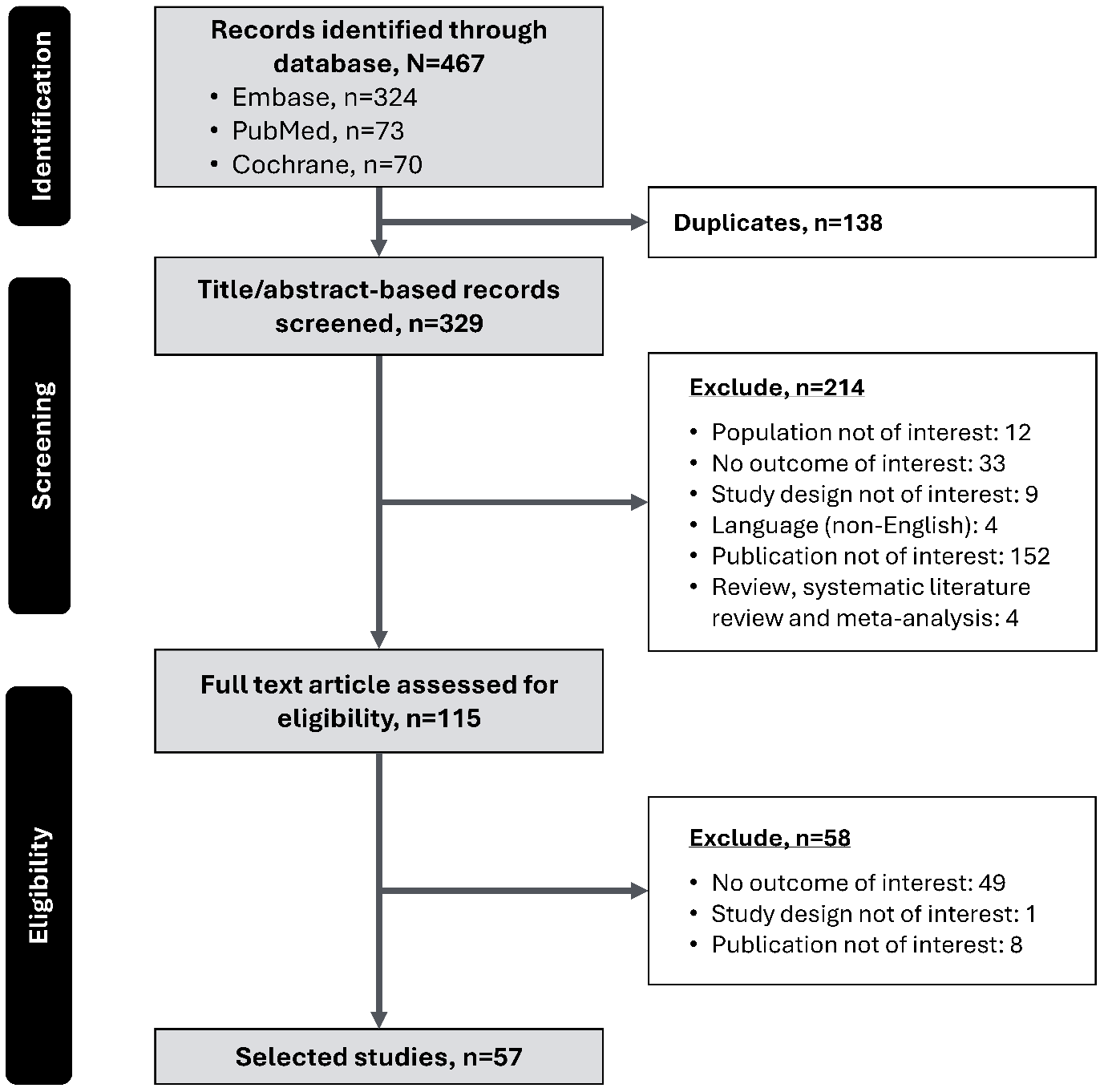


**eFigure 3.** Patient monitoring and belamaf dose adjustment from Cycle 1 to Cycle 5+
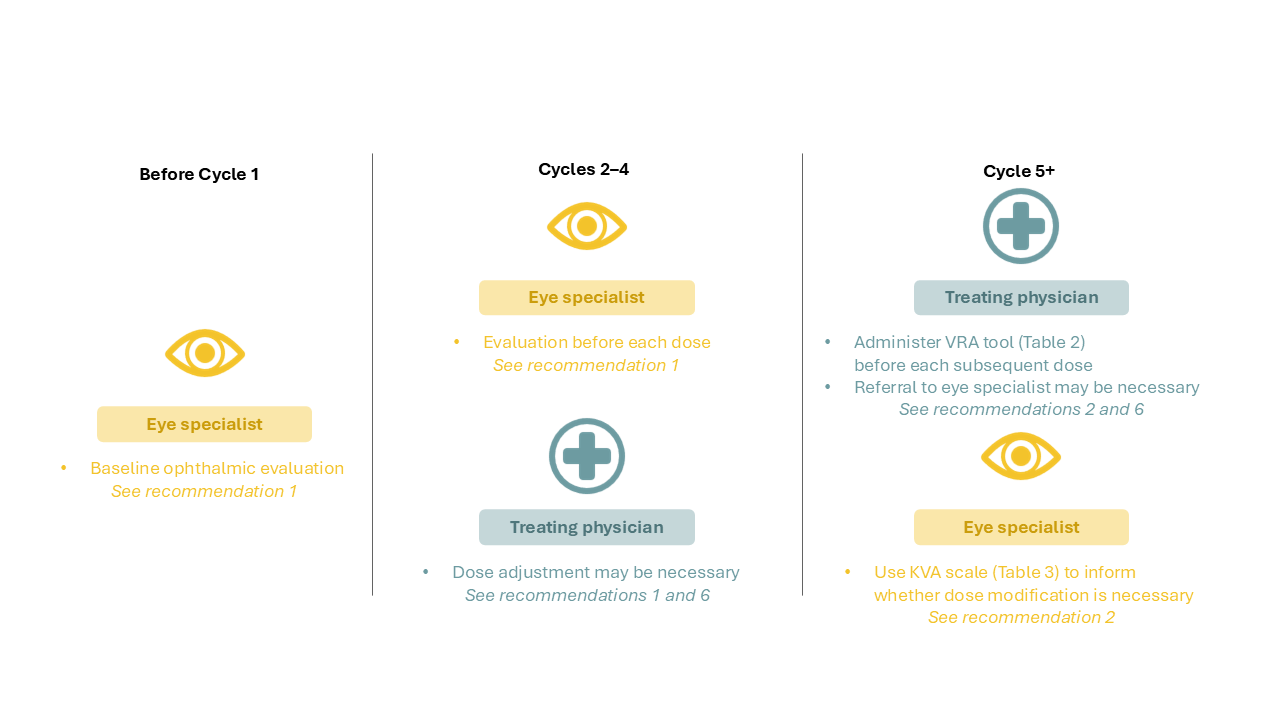


This figure provides an overview of the recommended process for belamaf therapy. For detailed guidance and considerations, please refer to the full recommendations in
Table 1.

KVA, Keratopathy and Visual Acuity; VRA, Vision-Related Anamnestic.
